# Diffusion of Galvanic Currents in the Human Organism

**Published:** 1889-04

**Authors:** 


### *DIFFUSION OF GALVANIC CURRENTS IN THE HUMAN ORGANISM.*

At the meeting of the Academy of Sciences in Paris, January 14th, M. Danion made a report of his studies in the diffusion of galvanic currents in the human system. He found that outside the skin and bones the various tissues of the organism have practically the same electric conductability: that of the bones is less by two-thirds than that of the other hypodermic tissues. In proportion as the electrodes, are brought together, the field of diffusion is limited, even to rendering it unnoticeable. It is necessary in practice to use large electrodes unless one wishes to secure superficial effects. The osseous structures cause an increase of resistance in proportion to their nearness to the surface; hence, the brain and cord are protected particularly from diffusion.—*Le Progès Mèdical*, January 26, 1889.

